# Virulence structure and its genetic diversity analyses of *Blumeria graminis* f. sp. *tritici* isolates in China

**DOI:** 10.1186/s12862-019-1511-3

**Published:** 2019-09-18

**Authors:** Xian Xin Wu, Xiao Feng Xu, De Xin Ma, Rong Zhen Chen, Tian Ya Li, Yuan Yin Cao

**Affiliations:** College of Plant Protection, Shenyang Agricultural University, Liaoning, Shenyang, +086110866 China

**Keywords:** *Blumeria graminis* f. sp. *tritici*, Genetic diversity, Virulence diversity, EST-SSR, Wheat powdery mildew, *Pm* gene

## Abstract

**Background:**

*Blumeria graminis* f. sp. *tritici* (*Bgt*), the causal agent of wheat powdery mildew severely affects yield security wheat production in China. Understanding the virulence structure and genetic variations of this pathogen is important for breeding wheat lines resistant to wheat powdery mildew. However, information related to genes controlling resistance remains elusive. This study analyzes the virulence structure and the genetic diversity of pathogenic *Bgt* populations isolated from northeastern (Liaoning, Heilongjiang) and northwestern (Gansu) China, two representative wheat producing areas, on 37 wheat cultivars each carrying a known powdery mildew resistance (*Pm*) gene.

**Results:**

*Bgt* isolates from northeastern China show higher frequencies of virulence genes than populations from Gansu Province. Many of the known *Pm* genes failed to provide resistance in this study. However, *Pm21* provided 100% resistance to all isolates from all three provinces, obtained during two consecutive years, while *Pm13* provided 100% resistance in Gansu. *Pm13*, *Pm16*, *Pm18*, and *Pm22* also showed partial resistance in northeastern China, while *Pm16*, *Pm18*, *Pm22*, *Pm5 + 6* and *Pm2 + 6 +*? maintained some resistance in Gansu. Genetic diversity among populations in different regions was detected by cluster analyses using expressed sequence tag-simple sequence repeat (EST-SSR). When the genetic similarity coefficient is relatively high, populations from the same regional origin are mostly clustered into one group while populations from different regions exhibit large genetic differences.

**Conclusion:**

*Pm21* remains the best choice for breeding programs to maintain resistance to *Bgt.* Only 58% of the isolates tested show a clear correlation between EST-SSR genetic polymorphisms and frequency of virulence gene data.

## Background

Wheat is a staple food for the majority of the world’s population. China accounted for 11.1% of the global wheat production in 2017, harvesting 134.33 million metric tons [1]. Powdery mildew, caused by *Blumeria graminis* f. sp. *tritici* (*Bgt*)*,* is one of the several fungal diseases seriously affecting wheat production [2]. This pathogen has a complex population structure, diverse physiological isolates, and extremely fast mutation rates [3]. The introduction of resistant cultivars increases selection pressure on pathogen populations, thus accelerating the emergence of new predominant pathogenic types and inducing the rapid accumulation of potential isolates, which results in the loss of resistance in cultivars. Therefore, analyzing population dynamics and genetic evolution, studying the spread and exchange of pathogens among various regions, and determining the variation of pathogenic isolates can allow scientists to continuously monitor the effectiveness of resistance genes and warn of the loss of resistance in cultivars as early as possible, so that adequate control measures can be proposed [4, 5]. Virulence frequency analysis has been used since the 1970s in monitoring population dynamics of wheat powdery mildew worldwide [6, 7], and has been widely used since the 1990s [8].

During the last 20 years, virulence monitoring of *Bgt* has been carried out continuously [9–12]*.* Numerous studies have been reported using molecular marker technologies in dissecting the genetic diversity of plant pathogens [13–15]. Several methods including the random amplification of polymorphic DNA (RAPD) [16], simple sequence repeat (SSR) [17], inter-simple sequence repeat (ISSR) [11], amplified fragment length polymorphism (AFLP) [18], and other molecular marker techniques have been used in studies of the genetic diversity of *Bgt* [11]. Moreover, Zhu et al. compared the virulence diversity and genetic polymorphism of *Bgt* and found no close correlation between these two factors [19]. Currently, most of these types of molecular markers can be used to reveal the polymorphism of genomic DNA. Nevertheless, the virulence phenotype of isolates on different host differentials is based on the gene expression level. However, few reports related to genetic polymorphism of *Bgt* from the perspective of gene expression sequences have been documented while no comparative analysis for the genetic diversity of expression sequences and virulence diversity have been carried out in China. In this study, in order to understand the effective protection provided by powdery mildew resistance (*Pm*) genes in northeastern China and explore the relationship between genetic polymorphism based on expression sequences and virulence diversity, the virulence of *Bgt* from northeastern China and from Gansu Province was monitored. The expressed sequence tag (EST)-SSR marker developed by Xu were applied to the analysis of the genetic diversity of *Bgt* and its regional association [20]. At the same time, the possible relationship between genetic diversity revealed by EST-SSR and virulence diversity was investigated.

## Results

### Frequency of virulence genes of *Bgt*

A total of 120 purified pathogenic isolates of *Bgt* were obtained from Liaoning, Heilongjiang and Gansu provinces during 2013–2014 (Additional file 1: Table S1). The frequency of virulence genes in these isolates was tested on 37 differentials, each carrying a known *Pm* gene. Occurrence frequencies of virulence genes in the various isolates ranged from 0 to 100%, while the susceptibility control Funo displayed a frequency of 100% (Table 1). The occurrence frequency of virulent virulence genes *V13*, *V16*, *V18* (*V1c*), *V21*, and *V22* (*V1e*) was less than 30.8% (Table 1). Therefore, their corresponding resistance genes, *Pm13*, *Pm16*, *Pm18* (*Pm1c*), *Pm21,* and *Pm22* (*Pm1e*) exhibited good resistance and are good resources for further breeding programs, especially *Pm21* which yielded 100% resistance to all the isolates. Therefore, *Pm21* was the most promising resistance gene against isolates from northeastern China which were collected in the period 2013–2014.

**Table 1:** Occurrence frequency of virulence genes in *Blumeria graminis* f. sp. *tritici* in 2013–2014

Cultivar (line)	Isogene	Virulence gene	Occurrence frequency of virulence genes (%)				
			2013		2014		
			Liaoning	Heilongjiang	Liaoning	Heilongjiang	Gansu
Axminster/8 cc	*Pm1*	*V1*	88.1	57.1	92.3	100	85.7
Ulka/8 cc	*Pm2*	*V2*	9.5	14.3	80.8	79.2	78.6
Asosan/8 cc	*Pm3a*	*V3a*	76.2	35.7	80.8	75.0	64.3
Chul/8 cc	*Pm3b*	*V3b*	23.8	85.7	46.2	75.0	35.7
Sonora/8 cc	*Pm3c*	*V3c*	95.2	92.9	92.3	100	100
Kolibri	*Pm3d*	*V3d*	76.2	71.4	65.4	83.3	85.7
W150	*Pm3e*	*V3e*	97.6	78.6	76.9	100	85.7
MichiganAmber/8 cc	*Pm3f*	*V3f*	97.6	64.3	80.8	83.3	92.9
Khapli/8 cc	*Pm4a*	*V4a*	85.7	21.4	57.7	66.7	92.9
Armada	*Pm4b*	*V4b*	81.0	0.0	80.8	70.8	85.7
Hope/8 cc	*Pm5*	*V5*	88.1	92.9	69.2	100	78.6
Tingalen	*Pm6*	*V6*	57.1	42.9	65.4	100	64.3
Coker747	*Pm6*	*V6*	92.9	100	73.1	91.7	85.7
CI14189	*Pm7*	*V7*	90.5	100	80.8	100	78.6
Kavkaz	*Pm8*	*V8*	92.9	78.6	61.5	66.7	61.4
R4A	*Pm13*	*V13*	14.3	7.1	23.1	28.3	14.3
Brigand	*Pm16*	*V16*	7.1	28.6	30.8	25.0	0.0
Amigo	*Pm17*	*V17*	–	–	76.9	100	42.9
MIN	*Pm18(Pm1c)*	*V18(V1c)*	28.6	21.4	19.2	8.3	7.1
XX186	*Pm19*	*V19*	–	–	88.5	91.7	64.3
KS93WGRC28	*Pm20*	*V20*	57.1	0.0	76.9	58.3	92.9
Yangmai 5/sub.6v	*Pm21*	*V21*	0.0	0.0	0.0	0.0	0.0
Virest	*Pm22(Pm1e)*	*V22(V1e)*	0.0	0.0	11.5	8.3	7.1
Line81–7241	*Pm23*	*V23*	59.5	21.4	65.4	58.3	92.9
Chiyacao	*Pm24*	*V24*	69.0	50.0	73.1	91.7	78.6
5P27	*Pm30*	*V30*	85.7	14.3	84.6	58.3	100
Kenguia	*Pm4 + 8*	*V4 + 8*	90.5	35.7	80.8	54.2	71.4
Coker983	*Pm5 + 6*	*V5 + 6*	26.2	57.1	30.8	41.7	21.4
Mission	*Pm4b + mli*	*V4b + mli*	28.6	14.3	73.1	70.8	85.7
Maris Dire	*Pm2 + mld*	*V2 + mld*	7.1	0.0	53.8	29.2	35.7
Normandie	*Pm1 + 2 + 9*	*V1 + 2 + 9*	92.9	57.1	76.9	95.8	100
Xiaobaidongmai	*PmXBD*	*VXBD*	14.3	35.7	26.9	33.3	35.7
Baimian 3	*Pm4 + 2X*	*V4 + 2X*	81.0	21.4	73.1	75.0	64.3
CI12632	*Pm2 + 6*	*V2 + 6*	35.7	78.6	46.2	62.5	42.9
Maris Huntsman	*Pm2 + 6 +*?	*V2 + 6 +*?	0.0	0.0	69.2	29.2	21.4
Era	*Era*	*Era*	9.5	0.0	61.5	33.3	35.7
Funo	*–*	*–*	100	100	100.0	100	100

In Gansu Province, resistance genes including *Pm13*, *Pm16*, *Pm18* (*Pm1c*), *Pm21*, *Pm22* (*Pm1e*), *Pm5 + 6* and *Pm2 + 6 +*? showed effective resistance against isolates from Gansu Province with infection scores from 0 to 2. *Pm16* and *Pm21* were the most effective and resistant genes and were immune to all tested isolates (Table 1).

### EST-SSR analysis

**Fig. 1:**
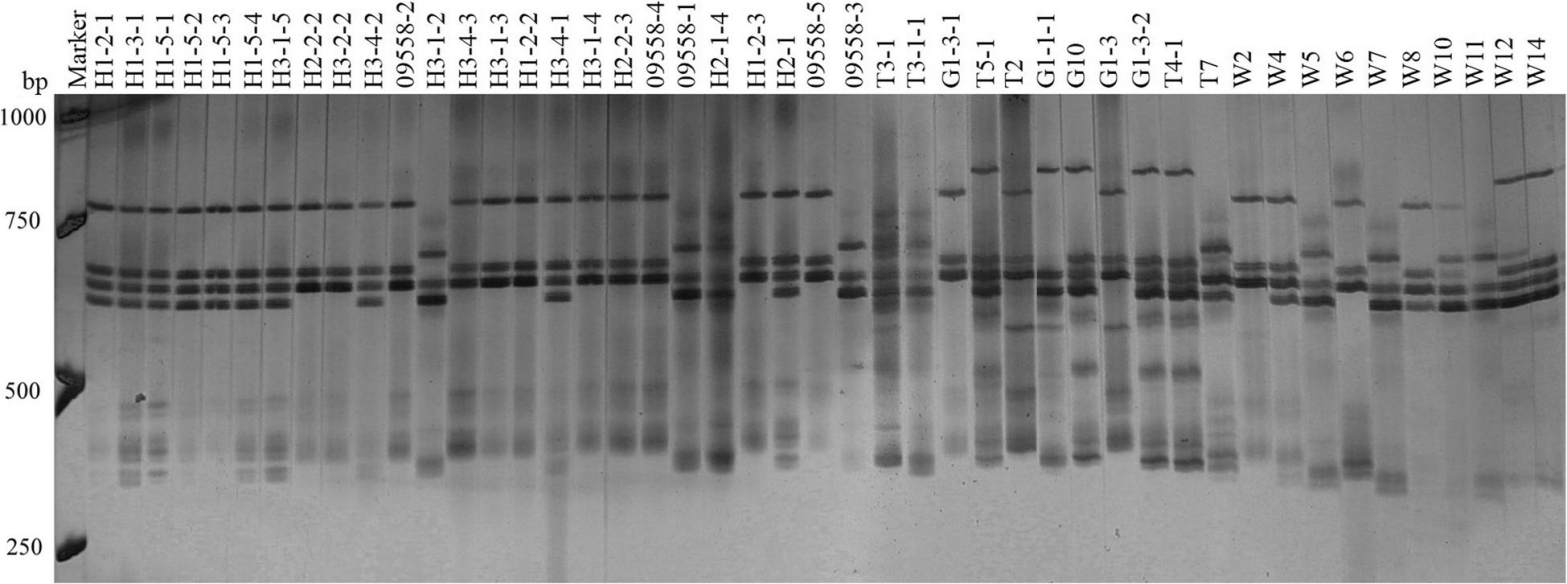
Representative PAGE image of PCR products amplified by marker Blu SSR3–1-Blu SSR3–2 using *B*. *graminis* f. sp. *tritici* DNA

**Fig. 2:**
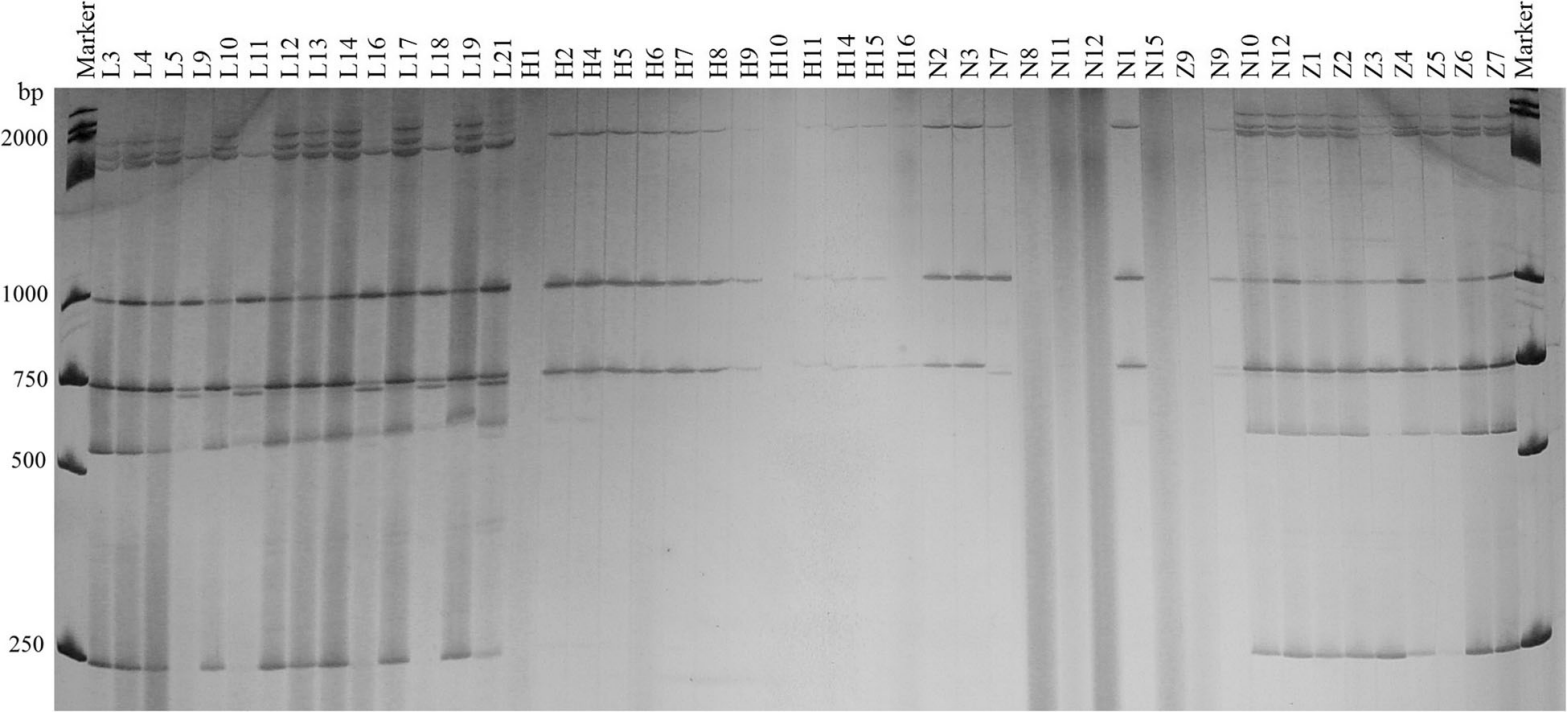
Representative PAGE image of PCR products amplified by marker Blu SSR29–1-Blu SSR29–2 using *B*. *graminis* f. sp. *tritici* DNA

Two pairs of EST-SSR primers, namely Blu SSR3–1-Blu SSR3–2 and Blu SSR29–1-Blu SSR29–2 which amplified clear and stable polymorphic bands, were chosen from among 7 reported EST-SSR primer pairs (Tabel 2) that were specific to *Bgt* isolated from northeastern China and Gansu Province. Figures 1 and 2 show the results of polyacrylamide gel electrophoresis (PAGE) using primer pairs Blu SSR3–1- plus Blu SSR3–2 and Blu SSR29–1- plus Blu SSR29–2, respectively.

### Genetic similarity analysis of *B. graminis* f. sp. *tritici* in different regions

**Fig. 3:**
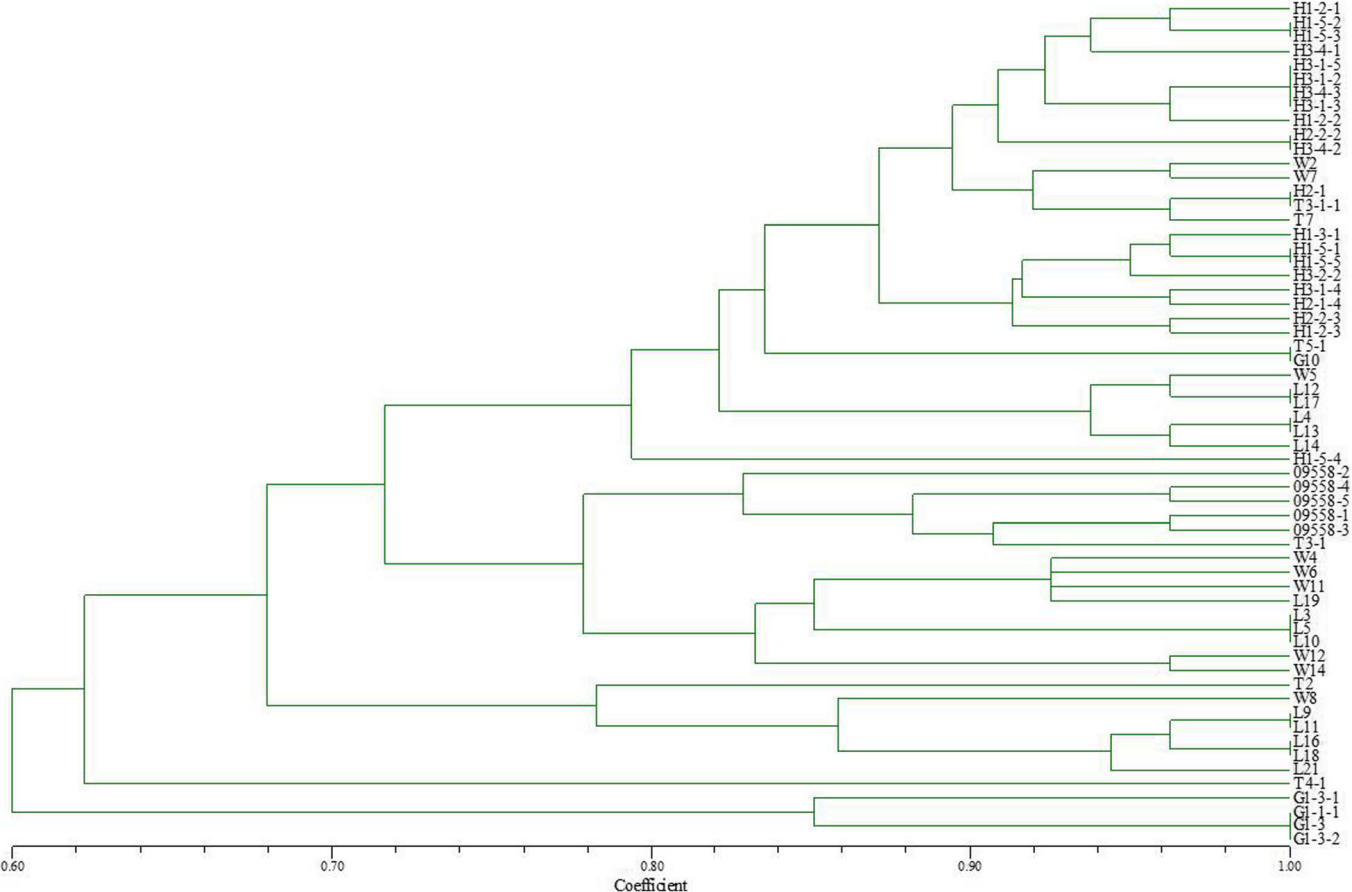
Dendrogram of clustering analysis based on EST-SSR data for the genetic diversity of *Blumeria graminis* f. sp. *tritici* isolates from different origins

Using the PAGE results of EST-SSR, polymorphism (Figs. 1 and 2), the genetic similarity of 60 isolates collected from Liaoning, Heilongjiang and Gansu provinces in 2014 was analyzed using NTSYSpc 2.10. The 60 isolates included 23 from Liaoning (strain numbers beginning with W and L), 26 from Heilongjiang (strain number beginning with H and 09558) and 11 from Gansu (strain number beginning with G and T). The clustering results based on genetic similarity coefficients are presented in Fig. 3.

When the genetic similarity coefficient was 0.82, T4.1 and T2 from Gansu and H1–5-4 from Heilongjiang were clustered individually. With the same similarity coefficient, the remaining 57 isolates were clustered into five groups. Group 1 contained 32 isolates, including 20 isolates from Heilongjiang, eight from Liaoning and four from Gansu. Group 2 consisted of six isolates, including five from Heilongjiang and one from Gansu. Group 3 and 4 were composed of nine and six isolates from Liaoning, respectively. Group 5 contained four isolates from Gansu. Moreover, based on a similarity coefficient of 0.90 the first group could be divided into five sub-groups as follows: the first sub-group consisted of 11 isolates from Heilongjiang; the second sub-group consisted of two isolates from Liaoning, two from Gansu and one from Heilongjiang; the third, fourth and fifth sub-group consisted of eight Heilongjiang isolates, two Gansu isolates and six Liaoning isolates, respectively. There may be some degree of genetic exchange through sexual reproduction among the *Bgt* from different provinces of the northeast and northwest regions. However, at a higher genetic similarity coefficient, the tested isolates were mostly clustered into groups by region, indicating that genetic differences were greatest between *Bgt* populations isolated from different geographical areas and smallest in *Bgt* populations isolated from the same area.

### Comparison analysis for genetic diversity and virulence diversity of wheat powdery mildew

**Fig. 4:**
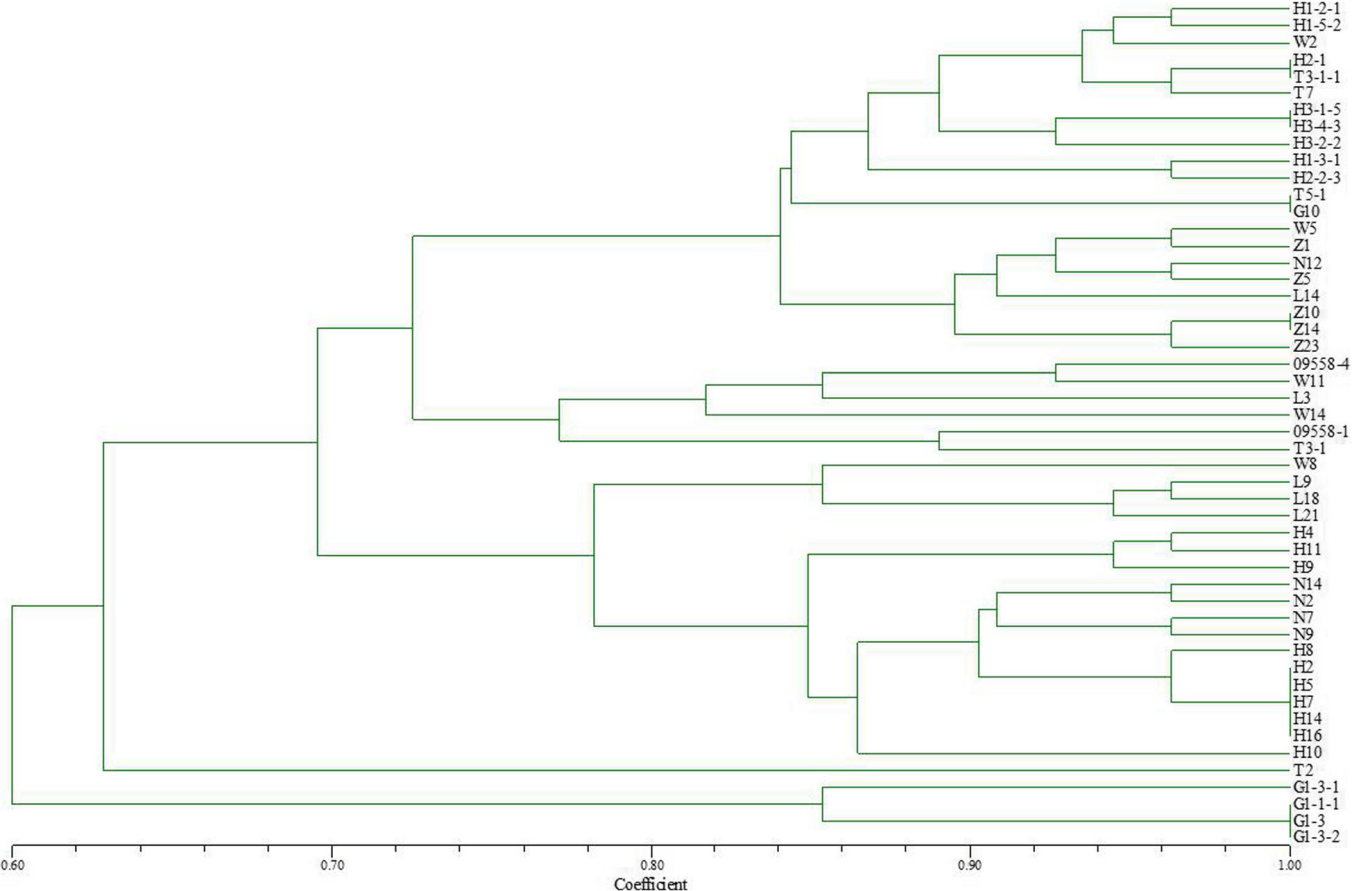
Clustering analysis based on EST-SSR data for the genetic diversity of 50 *Blumeria graminis* f. sp. *tritici* isolates from different origins

According to the EST-SSR polymorphisms highlighted by PAGE of 50 isolates, a phylogenetic tree was constructed using genetic NTSYSpc 2.10 (Fig. 4). As can be concluded, 49 isolates, at 0.765 similarity coefficient, (except for T2 from Gansu that clustered into a single group), were naturally clustered into four groups. The first group consisted of 21 isolates, including eight isolates from Heilongjiang in 2014, six isolates from Liaoning in 2013, three isolates from Liaoning in 2014 and four isolates from Gansu in 2014. The second group consisted of three isolates from Liaoning, two isolates from northern Heilongjiang and one strain from Gansu in 2014. The third group consisted of 18 isolates, including four isolates from Liaoning, ten from Heilongjiang in 2013 and four from Liaoning in 2014. The fourth group was composed of four isolates from Gansu Province in 2014.

When the similarity coefficient of polymorphism is 0.86, the first large group can be subdivided into three subgroups. Subgroup I includes eight isolates (H1–2-1, H1–5- 2, H2–1, H3–1-5, H3–4-3, H3–2-2, H1–3-1, and H2–2-3) collected from Heilongjiang, one (W2) from Liaoning and two (T3–3-1, and T7) from Gansu in 2014. Subgroup II includes two isolates (T5–1, and G10) collected from Gansu. Subgroup III includes eight isolates from Liaoning of which two (W5, and L14) were collected in 2014 and six (Z1, Z5, Z10, Z14, Z23, and N12) were collected in 2013. When the similarity coefficient was increased to 0.90 (the third large category) in addition to W8 and H10, the rest of the isolates were clustered into three subgroups. Subgroup I includes 3 isolates (L9, L18, and L21) from Liaoning in 2014. Subgroup II includes three isolates (H4, H9, and H11) from Heilongjiang in 2013. Subgroup III includes four (N2, N7, N9, and N14) from Liaoning and six (H2, H5, H7, H8, H14, and H16) from Heilongjiang in 2013.

A ‘0, 1’ matrix for the virulence diversity of the 50 isolates was built based on the infection types of those 50 *Bgt* isolates on 37 differential hosts. Then, a phylogenetic tree was constructed according to the similarity of the infection effect on the hosts (Fig. 4).

**Fig. 5:**
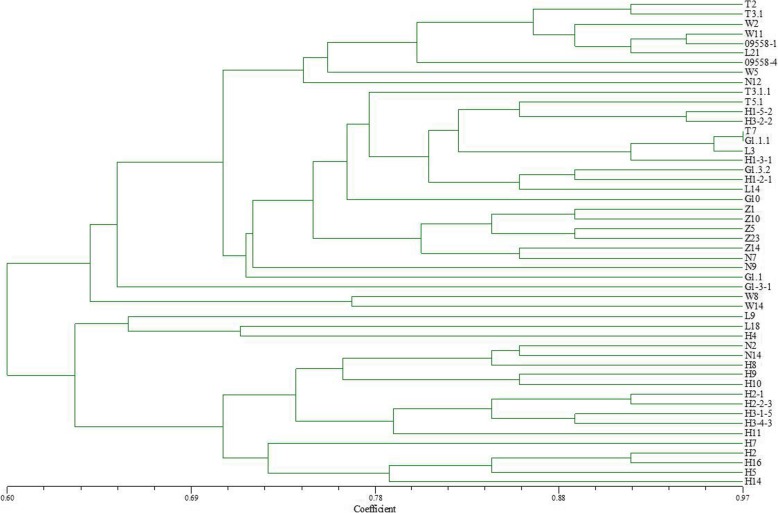
Clustering analysis for the virulence diversity of 50 *Blumeria graminis* f. sp. *tritici*

The 50 tested isolates were clustered according to their virulence diversity, although the overall similarity coefficient was relatively low (Fig. 5). When the similarity coefficient was 0.627, the isolates were clustered into two major groups. However, when the similarity coefficient was increased to 0.753, N12, and N9 from Liaoning in 2013, L9, and L18 from Liaoning in 2014, H4, and H7 from Heilongjiang in 2013, and G1.1, G1–3-1 from Gansu in 2014, were individually clustered in a group with relatively low similarity when compared with the other isolates in base on virulence diversity. The remaining 42 isolates were naturally clustered into seven groups. The first group consisted of eight isolates, including two (T2, and T3.1) isolates from Gansu in 2014, four (W2, W5, W11, and L21) from Liaoning 2014, and two (09558–1, and 09558–4) from northern Heilongjiang in 2014. The second group consisted of 12 isolates collected in 2014, including six isolates (T3–1-1, T5–1, T7, G1–1-1, G1–3-2, and G10) from Gansu, four (H1–5-2, H3–2-2, H1–3-1, and H1–2-1) from Heilongjiang and two (L3, and L14) from Liaoning. The third group was composed of six isolates (Z1, Z5, Z10, Z14, Z23, and N7) from Liaoning in 2013. The fourth group consisted of two isolates (W8, and W14) from Liaoning in 2014. The fifth group consisted of two isolates (N2, and N14) from Liaoning and three (H8, H9, and H10) from Heilongjiang, all in 2013. The sixth group consisted of five isolates from Heilongjiang, including four (H2–1, H2–2-3, H3–1-5, and H3–4-3) collected in 2014 and one (H11) collected in 2013. The seventh group included four isolates (H2, H5, h14, and H16) from Heilongjiang in 2013.

The clustering figures of EST-SSR polymorphism (Fig. 4) and virulence diversity (Fig. 5) were compared and when the similarity coefficient was higher than 0.75. Twenty-nine isolates were clustered into the same eight groups by the EST-SSR polymorphism and virulence diversity in combination. Group 1 was composed of N2 and N14 while Group 2 included H8 and H10. Group 3 comprised T5–1 and G10. Group 4 consisted of H2, H5, H14, and H16. Group5 contained 09558–1, 09558–4, W11, and T3–1. Group 6 comprised Z2, Z5, Z10, Z14, and Z23. Group 7 was composed of H2–1, H2–2-3, H3–1-5, and H3–4-3. Group 8 included T3–1-1, T7, H1–2-1, H1–3-1, H1–5-2, and H3–2-2. The positions of the remaining isolates were from very different locations within the clustering figures of gene diversity and virulence diversity with no corresponding relationship. The results showed that a certain degree of correlation existed between the genetic polymorphisms and the virulence diversity in 29 out of 50 isolates tested (58%). However, for the remaining 21 isolates the EST-SSR polymorphisms did not correspond to the virulence diversity on the differentials with a one to one match.

## Discussion

### Virulence diversity of *Bgt* in northeastern China

Virulence frequency determination, based on the gene-for-gene hypothesis and used in relation to important pathogens such as powdery mildew [10, 21, 22], rust [23], rice blast [24], is a helpful tool to provide a theoretical basis for disease prevention and control. Unfortunately, due to the variation of virulence genes in pathogenic fungi resistance genes often lose their efficacy rather quickly after being introduced in practice [25], because when a resistant cultivar becomes widely popular and is heavily grown by producers, its new status results in the exertion of selection pressure on the original pathogenic group. The virulent fungal group will gradually propagate, accumulate and spread to become the dominant microbial group. The original dominant fungal group loses its optimal host conditions and becomes the vulnerable fungal group. In this process, some of the previously moderately or weakly virulent fungi will be eliminated during this selective evolution, while others may mutate into more virulent strains to adapt to the current resistant cultivars. When the dominant fungal group reaches the status of being the absolute dominant fungal group, the wheat cultivar loses its resistance and becomes susceptible to the new fungal race. *Pm8*-*Yr9*-*Lr26*-*Sr31*, a linkage gene cluster derived from wheat-rye 1BL/1RS chromosome translocation line, is widely used in wheat breeding because of its high resistance to wheat stem rust, stripe rust, leaf rust and powdery mildew [26, 27]. However because of overuse of this gene clusters resistance gene *Pm8* lost its efficacy to control powdery mildew in a period of 10 years [28]. In this study, the *Pm8* gene was basically ineffective against 120 powdery mildew isolates tested, with virulence frequencies over 61.4%. Gene *Pm21* is a broad-spectrum disease-resistant gene from *Triticum villosum*, which is widely used in wheat breeding worldwide because of its effective resistance to powdery mildew. *Pm21* has been proven to express immunity or near immunity to almost all powdery mildew isolates in China as well as to 120 European physiological races [29–31].

Consistent with previous studies, no isolate was found to be virulent to *Pm21* in this study. The frequency of the virulence gene V21 in both in northeastern China and Gansu is 0% and its corresponding resistance gene *Pm21* continues to be effective, and currently provides the best genetic resistance to wheat powdery mildew. Recently, Wang (2017) analyzed the virulence of 434 *Bgt* strains collected from 16 provinces in China from 2015 to 2016. The results showed that the virulence frequencies of *Bgt* to *Pm12*, *Pm13*, *Pm16*, *Pm21* and *Pm35* were less than 30%, of which *Pm12* were less than 5%, and no isolates were virulent to *Pm21* [32]. In addition, we analyzed the virulence of *Bgt* strains collected annually from northeast China from 2015 to 2018, and found no isolates were virulent to *Pm21* (unpublished). Prolonging the resistance duration of *Pm21* gene can be achieved by enriching the diversity of resistance genes by utilizing other effective resistance genes in wheat breeding. Continuous studies including our research in 2008–2014 identified that apart from *Pm21*, the resistance genes *Pm13*, *Pm16*, *Pm18*, and *Pm22* also showed effective resistance to powdery mildew, and can be combined with *Pm21* to enhance and extend the resistance of varieties [33]. Moreover, most of these effective resistance genes are stable and a relatively small number of these genes’ resistance has changed. Therefore, continuous virulence monitoring of *Bgt* can provide a reliable basis for breeding resistance to wheat powdery mildew. Our results also show that although there were many common virulent genes among different geographical groups, the frequency of some virulent genes varied greatly among different regions. For instance, the frequency of V16 was 0% in Gansu vs. 30.8% in Liaoning and 25.0% in Heilongjiang. These differences and characteristics from different regions provide key guidance for reasonable rotation and distribution of wheat varieties, thus effectively reducing the occurrence of powdery mildew.

### *Bgt* clustering analysis for gene polymorphism

According to the clustering results for gene polymorphisms in isolates from Liaoning, Heilongjiang and Gansu provinces, some of the isolates from different regions were found to be clustered together in a group, indicating that *Bgt* in these three provinces may have a certain level of genetic exchange. However, when the genetic similarity coefficient is relatively high, isolates from different locations are basically clustered into separate groups by region showing that strain populations from different regions exhibit large genetic differences. Years of research have shown that in China pathogens of airborne diseases such as wheat stem rust, and powdery mildew mainly rely on monsoon to spread throughout the country. In spring, pathogens of airborne diseases spreads from south to north through two route, one goes from Yunnan, Guizhou, Guangxi and other places across Sichuan to the spring wheat areas of Gansu and Xinjiang; the other goes from the southern wheat areas such as Fujian and Guangxi across the wheat production areas along the southeast coast to Shandong, and finally spreads to the northeast wheat region. In autumn, the powdery mildew strains spread from the northern wheat region to the southern wheat region according to the reverse direction of the monsoon [19]. This completes the cycle of the exchange of strains between northern and southern regions. Therefore, strains from different regions have the opportunity for close encounters, leading to gene exchange, through sexual reproduction, resulting in new genotypes and virulence types, thus achieving gene exchange in different regions. Comparing EST-SSR polymorphism of genes with virulence polymorphism to differentials, we found that 29 of the 50 tested isolates were clustered together both in gene polymorphism and virulence features in eight groups with different combinations. This indicates that the gene expression sequence polymorphism of *Bgt* that was revealed by the EST-SSR molecular marker technique and the corresponding virulence diversity are correlated. However, the other 42% of the isolates display great differences in polymorphism and virulence diversity, without any correlation. This result is consistent with previous research results using other molecular marker technologies, such as RAPD [16, 34]; ISSR [20, 35]; AFLP [18]; SSR [17] and SNPs [36]. These molecular marker methods were all able to reveal abundant DNA polymorphism. However, the genetic polymorphism without one-to-one correspondence to virulence diversity, or without showing a certain correlation with virulence diversity was very small.

The gene expression sequence diversity is not characterized by a simple one-to-one correspondence with its virulence diversity to the host in the same strain. We propose that there may be several reasons: first, it is possible that one gene from the expression sequence controls many virulence traits of *Bgt*, or that multiple genes control one virulence trait together. Second, the internal interaction among genes of *Bgt*, and the interaction between genes of *Bgt* and host genes may also change biological traits. In addition, gene expression is influenced by environmental factors, and the virulence of *Bgt* in wheat is the result of the interaction of genotype and environmental conditions. In view of this, we believe that although EST-SSR and other molecular methods have the advantage of high efficiency and less interference in the dynamic early warning of *Bgt* and regional correlation analysis, it is not possible to rely solely on molecular methods because the gene sequence of the strain does not correspond well with its pathogenicity. A large number of single-resistance gene cultivar lines are needed to identify the host in the traditional virulence monitoring process relying on inoculation identification, which is time-consuming and laborious, in order to provide effective basic information for the rational prevention and control of wheat powdery mildew disease, disease resistance breeding and variety distribution. However, at present, single-gene line identification is still recommended to be the main method, supplemented by EST-SSR or other molecular markers.

## Conclusion

This study investigated and analyzed pathogenic *Bgt* populations from northeastern (Liaoning, Heilongjiang) and northwestern (Gansu) China, two representative wheat producing areas. The results indicate that *Bgt* populations from northeastern China showed higher frequencies of virulence than populations from Gansu Province. While many of the known resistance genes failed to provide resistance. *Pm21* provided 100% resistance to all the isolates from the three provinces and is therefore provided the best resistance. *Pm13*, *Pm16* (100% resistance in Gansu), *Pm18*, and *Pm22* also maintained acceptable levels of resistance in China. Populations of *Bgt* from different regions exhibit large genetic differences. The gene expression sequence polymorphisms of *Bgt* revealed by the EST-SSR molecular marker technique and the corresponding virulence diversity was shown to correlate in only 29 out of 50 isolated tested.

## Methods

### Collection and preparation of *Bgt* isolates from various regions in China during 2013–2014

To analyze regional differences in *Bgt* virulence, samples of wheat powdery mildew were collected from main growth areas of wheat in the Liaoning and Heilongjiang provinces in Northeastern China (Additional file 1: Table S1). Additionally, samples from Gansu Province were also collected from wheat powdery mildew and rust detection nurseries. The pre-prepared susceptible cultivar Chancellor was used for the isolation, purification, and maintenance of pathogenic isolates. Chancellor plants were first sown in earthen pots. When the primary leaves were fully expanded or when the seedlings were about seven days old, the leaves were removed from the seedlings and placed in 15 cm diameter petri dishes onto two layers of filter paper moistened with 40 mg·L^− 1^ 6-benzylaminopurine solution to increase the lifespan of the harvested leaves. Both ends of the detached leaves were immobilized with glass strips. Each individual isolate was selected and evenly smeared on the detached leaves using whittled-flat toothpicks. After inoculation, the petri dishes were placed in a growth chamber at 18–20 °C, 8000 Lx, and 14 h/10 h light/dark cycle. When white conidia appeared on the leaves (5 days after inoculation), isolation or purification of single pustules was conducted using standard procedures. The experiments were conducted in triplicates obtain a single pustule, and the single isolate was coded and kept for in vivo propagation [20].

### Virulence frequency analysis of *Bgt*

**Fig. 6:**
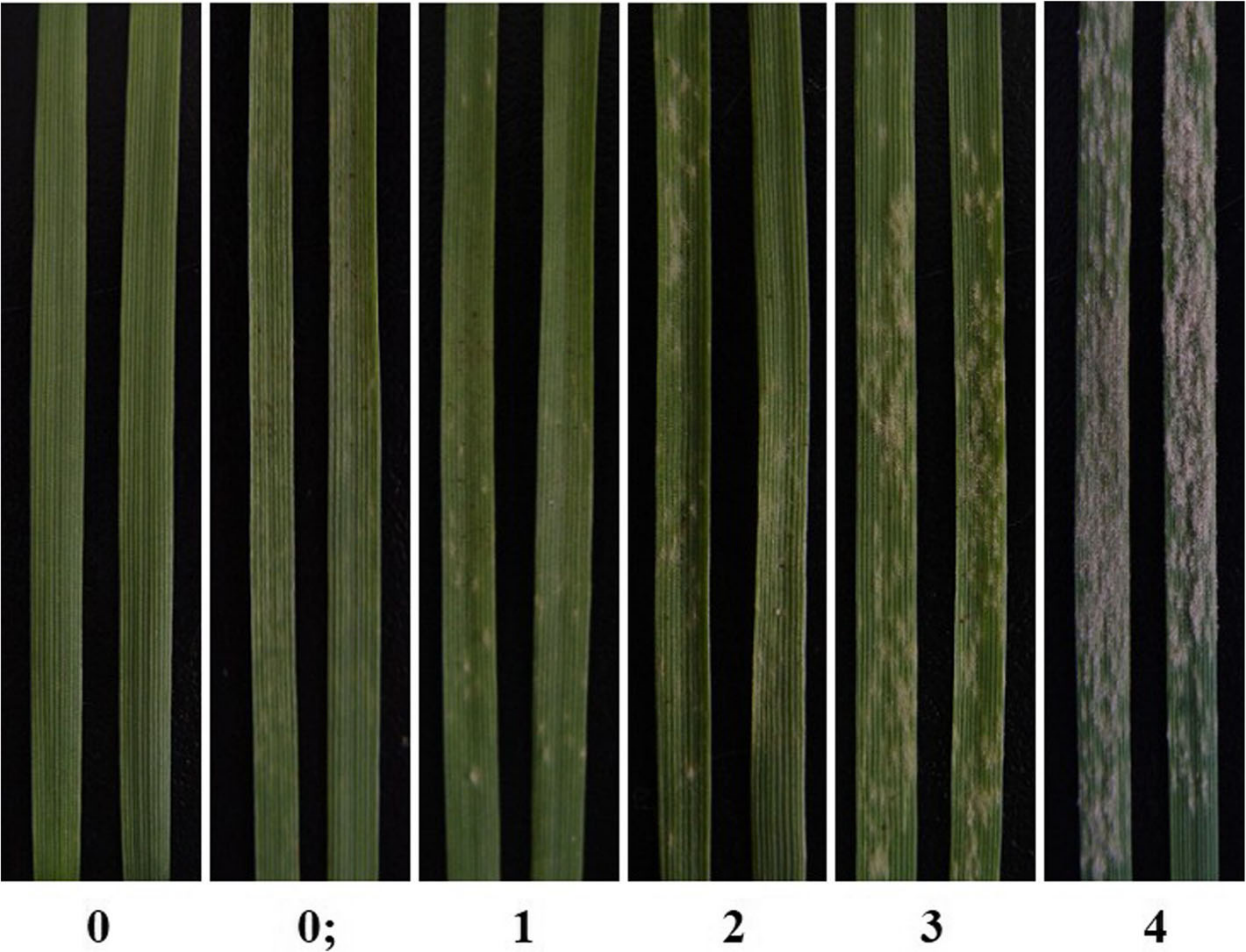
The infection types used in this study

Virulence frequency was analyzed in 37 isogenic lines, each harboring a single known powdery mildew resistance gene (Table 1). The thirty-seven differentials were sown in 10 cm diameter earthen pots, and marked clearly to identify each line. The susceptible cultivar Chancellor was used as a control. When the primary leaves were fully expanded (about 7 days), each purified *Bgt* isolate (as described above) was inoculated on all 37 differential isogenic lines. About 7 days later, when the Chancellor showed a clear level of susceptible symptoms, the infection types (ITs) of all the leaves were observed and recorded using the stringent evaluation standard of Si et al. [37]. The classification standard of reaction type was recorded using the 0–4 classification standard of Si et al. [37], namely ITs 0–2 recorded as ‘resistant’ (R) and their corresponding isolates are defined as avirulent; ITs 3–4 recorded as ‘susceptible’ (S) and their corresponding isolates were virulent (typical examples of ITs 0–4 are shown in Fig. 6). In addition, the frequency of occurrence of the corresponding genes for virulence in *Bgt* was calculated. To increase the accuracy of the results, each combination of isolate and cultivar was replicated three times.

### Genomic DNA extraction and polymerase chain reaction (PCR) analysis

Genomic DNA was extracted from conidia with an Omega Bio-Tek fungal DNA kit (Norcross, GA, USA) following the manufacturer’s protocol. The total PCR reaction volume was 20 μL, consisting of 10 μL 2 × Power *Taq* PCR Master Mix, 1 μL forward primer, 1 μL reverse primer, 1 μL DNA template (30 ng·μL^− 1^), 7 μL ddH_2_O. The PCR procedure was as follows: initial denaturation 5 min at 94 °C; then 35 cycles of: denaturation 30 s at 94 °C; annealing 45 s at the primer pair-specific annealing temperature (Table 2); extension 1.5 min at 72 °C; followed by a final extension for 10 min at 72 °C.

**Table 2:** Seven EST-SSR primer pairs suitable for analyzing the DNA polymorphism in Bgt

codes	Primers	Sequences	repeat motif	Annealing temperature (°C)
1-F	Blu SSR3–1	TTCGAGGCAAGCTCTTCTCA	(CCGTTC)_4_	56
1-R	Blu SSR3–2	TTTCGGCAGGCAAGTTTATT		
2-F 2-R	Blu SSR18–1	GGGTAACGATTGGTTAGGTGCT	(ATCACC)_3_	56
	Blu SSR18–2	AGGTGGTGGTAAAGGGGATGAT		
3-F 3-R	Blu SSR29–1	GGAGGATCGGTAGCAGTG	(GCA)_5_	56
	Blu SSR29–2	GCGGCGGTAGCTTCTTTT		
4-F	Blu SSR32–1	GGGGAGGTATAGGTGAGG	(TCT)_6_	52
4-R	Blu SSR32–2	GAGCGTTTGCTGTTCTGT		
5-F	Blu SSR35–1	AGACTCACAGCAGAGCAAA	(CTTCAA)_3_	52
5-R	Blu SSR35–2	GCAGATCCATGATCTTCGT		
6-F	Blu SSR41–1	ATCCATTGTAGTTAGGAGCCA	(AC)_6_	54
6-R	Blu SSR41–2	ATGACCTGATTGATTTATCCC		
7-F	Blu SSR44–1	TGAGGATTTAGATGATATGGA	(AGA)_5_	52
7-R	Blu SSR44–2	GATCTTAAATTATTTTGACCG		

### Selection of the EST-SSR primers

Seven pairs of EST-SSR primers (Table 2) were designed according to Xu [20], and were screened for occurrence of clear and stable polymorphisms. Two pairs were chosen for the genetic polymorphism analysis. The primers were synthesized by Sangon Biotech Inc. (http://www.sangon.com/, Shanghai, China).

### Polyacrylamide gel electrophoresis and genetic diversity analysis

The procedure for polyacrylamide gel electrophoresis (PAGE) was followed as previously published by Chen et al. [38]. The silver staining method used to visualize the PCR products here was described by Bassam et al. [7]. Based on the of PAGE results (Figs. 1 and 2), ‘1’ and ‘0’ were assigned to the presence or absence of bands, respectively, and the same method was used for frequency of virulence analysis, ‘1’ and ‘0’ were assigned to resistance (ITs: 0–2) or susceptibility (ITs: 3–4) in the host. According to the ‘1, 0’ data matrix, genetic similarity was calculated using NTSYSpc 2.10 statistical software. The unweighted pair group arithmetic method was used for gene diversity cluster analysis of the expression sequences and then the classification trees were constructed.

## Supplementary information

**Additional file 1: Table S1.** Collection dates, sites, and cultivars for all *Bgt* isolates used in this study (DOCX 29 kb)

## Data Availability

All data generated or analysed during this study are included in this published article.

## Abbreviations

AFLP: Amplified fragment length polymorphism
*Bgt*: *Blumeria graminis* f. sp. *tritici*
EST-SSR: Expressed sequence tag-simple sequence repeat
ISSR: Inter-simple sequence repeat
ITs: Infection types
PAGE: Polyacrylamide gel electrophoresis
*Pm*: Powdery mildew resistance genes
RAPD: Random amplification of polymorphic DNA
SNPs: Single nucleotide polymorphism
SSR: Simple sequence repeat
