# Supplementary material for: Virulence structure and its genetic diversity analyses of Blumeria graminis f. sp. tritici isolates in China
Source: BMC Evol Biol. 2019 Sep 18;19:183. doi: 10.1186/s12862-019-1511-3 (PMC6751876; doi:10.1186/s12862-019-1511-3)
Supplement: Supplementary file 1 — Additional file 1: Table S1. Collection dates, sites, and cultivars for all Bgt isolates used in this study (DOCX 29 kb) [file 12862_2019_1511_MOESM1_ESM.docx]

Additional file 1: Table S1. Collection dates, sites, and cultivars for all *Bgt* isolates used in this study

| Isolate | Collection date | Collection site | Cultivars |
| --- | --- | --- | --- |
| Z1 | 16-Jun-13 | Fuxin, Liaoning | Advanced Lines |
| Z2 | 16-Jun-13 | Fuxin, Liaoning | Advanced Lines |
| Z3 | 16-Jun-13 | Fuxin, Liaoning | Advanced Lines |
| Z4 | 16-Jun-13 | Fuxin, Liaoning | Liaochun 15 |
| Z5 | 16-Jun-13 | Fuxin, Liaoning | Liaochun 15 |
| Z6 | 16-Jun-13 | Fuxin, Liaoning | Liaochun 15 |
| Z7 | 15-Jun-13 | Shenyang, Liaoning | Little Club |
| Z8 | 15-Jun-13 | Shenyang, Liaoning | Little Club |
| Z9 | 15-Jun-13 | Shenyang, Liaoning | Trapping nursery |
| Z10 | 20-Jun-13 | Shenyang, Liaoning | Trapping nursery |
| Z11 | 20-Jun-13 | Shenyang, Liaoning | Volunteer |
| Z12 | 20-Jun-13 | Shenyang, Liaoning | Volunteer |
| Z13 | 20-Jun-13 | Shenyang, Liaoning | Volunteer |
| Z14 | 17-Jun-13 | Dongling, Liaoning | Chancellor |
| Z15 | 17-Jun-13 | Dongling, Liaoning | Chancellor |
| Z16 | 17-Jun-13 | Dongling, Liaoning | Chancellor |
| Z17 | 17-Jun-13 | Dongling, Liaoning | Shenmian 2137 |
| Z18 | 17-Jun-13 | Dongling, Liaoning | Shenmian 2137 |
| Z19 | 17-Jun-13 | Dongling, Liaoning | Shenmian 2135 |
| Z20 | 17-Jun-13 | Dongling, Liaoning | Shenmian 2135 |
| Z21 | 16-Jun-13 | Chaoyang, Liaoning | Volunteer wheat |
| Z22 | 16-Jun-13 | Chaoyang, Liaoning | Volunteer wheat |
| Z23 | 16-Jun-13 | Chaoyang, Liaoning | Liaochun 25 |
| Z24 | 16-Jun-13 | Chaoyang, Liaoning | Liaochun 25 |
| Z25 | 16-Jun-13 | Chaoyang, Liaoning | Liaochun 25 |
| Z26 | 16-Jun-13 | Chaoyang, Liaoning | Local variety, breeding parent nursery |
| Z27 | 16-Jun-13 | Chaoyang, Liaoning | Local variety, breeding parent nursery |
| Z28 | 16-Jun-13 | Chaoyang, Liaoning | Local variety, breeding parent nursery |
| Z29 | 16-Jun-13 | Chaoyang, Liaoning | Local variety, breeding parent nursery |
| N1 | 15-Jun-13 | Liaoyang, Liaoning | Liaochun 25 |
| N2 | 15-Jun-13 | Liaoyang, Liaoning | Liaochun 25 |
| N3 | 15-Jun-13 | Liaoyang, Liaoning | Wheat landrace |
| N4 | 15-Jun-13 | Liaoyang, Liaoning | Wheat landrace |
| N5 | 17-Jun-13 | Xinmin，Liaoning | Liaochun 9 |
| N6 | 17-Jun-13 | Xinmin，Liaoning | Liaochun 9 |
| N7 | 17-Jun-13 | Xinmin，Liaoning | Liaochun 9 |
| N8 | 17-Jun-13 | Xinmin，Liaoning | Trapping nursery |
| N9 | 17-Jun-13 | Xinmin，Liaoning | Trapping nursery |
| N10 | 17-Jun-13 | Xinmin，Liaoning | Trapping nursery |
| N11 | 17-Jun-13 | Anshan, Liaoning | Little Club nursery |
| N12 | 17-Jun-13 | Anshan, Liaoning | Little Club nursery |
| N14 | 17-Jun-13 | Anshan, Liaoning | Liaochun 9 |
| N15 | 17-Jun-13 | Anshan, Liaoning | Liaochun 9 |
| H1 | 17-Jun-13 | Harbin, Heilongjiang | Trapping nursery |
| H2 | 17-Jun-13 | Harbin, Heilongjiang | Trapping nursery |
| H3 | 17-Jun-13 | Harbin, Heilongjiang | Volunteer wheat |
| H4 | 17-Jun-13 | Harbin, Heilongjiang | Volunteer wheat |
| H5 | 18-Jun-13 | Jiusan, Heilongjiang | Advanced Lines |
| H6 | 18-Jun-13 | Jiusan, Heilongjiang | Advanced Lines |
| H7 | 18-Jun-13 | Jiusan, Heilongjiang | Chancellor |
| H8 | 18-Jun-13 | Jiusan, Heilongjiang | Chancellor |
| H9 | 18-Jun-13 | Keshan, Heilongjiang | Volunteer wheat |
| H10 | 18-Jun-13 | Keshan, Heilongjiang | Volunteer wheat |
| H11 | 18-Jun-13 | Keshan, Heilongjiang | Volunteer wheat |
| H14 | 18-Jun-13 | Beian, Heilongjiang | Afu |
| H15 | 18-Jun-13 | Beian, Heilongjiang | Afu |
| H16 | 18-Jun-13 | Beian, Heilongjiang | Afu |
| W1 | 18-Jun-14 | Fuxin, Liaoning | Shenmian 2135 |
| W2 | 18-Jun-14 | Fuxin, Liaoning | Little Club nursery |
| W4 | 18-Jun-14 | Fuxin, Liaoning | Little Club nursery |
| W5 | 18-Jun-14 | Chaoyang, Liaoning | breeding parent nursery |
| W6 | 18-Jun-14 | Chaoyang, Liaoning | breeding parent nursery |
| W7 | 18-Jun-14 | Chaoyang, Liaoning | breeding parent nursery |
| W10 | 17-Jun-14 | Xinmin，Liaoning | Trapping nursery |
| W11 | 17-Jun-14 | Xinmin，Liaoning | Trapping nursery |
| W12 | 17-Jun-14 | Xinmin，Liaoning | Liaochun 9 |
| W14 | 17-Jun-14 | Xinmin，Liaoning | Liaochun 9 |
| L3 | 19-Jun-14 | Liaoyang, Liaoning | Liaochun 25 |
| L4 | 19-Jun-14 | Liaoyang, Liaoning | Liaochun 25 |
| L5 | 19-Jun-14 | Liaoyang, Liaoning | Chiyacu, Trapping nursery |
| L6 | 19-Jun-14 | Liaoyang, Liaoning | Chiyacu, Trapping nursery |
| L9 | 19-Jun-14 | Liaoyang, Liaoning | Chiyacu, Trapping nursery |
| L10 | 16-Jun-14 | Dongling, Liaoning | Little Club nursery |
| L11 | 16-Jun-14 | Dongling, Liaoning | Little Club nursery |
| L12 | 16-Jun-14 | Dongling, Liaoning | Mingxian 169 (Trapping nursery) |
| L13 | 16-Jun-14 | Dongling, Liaoning | Mingxian 169 (Trapping nursery) |
| L14 | 16-Jun-14 | Dongling, Liaoning | Chancellor |
| L16 | 16-Jun-14 | Shenyang, Liaoning | Chinese Spring |
| L17 | 16-Jun-14 | Shenyang, Liaoning | Chinese Spring |
| L18 | 16-Jun-14 | Shenyang, Liaoning | Chancellor |
| L19 | 16-Jun-14 | Shenyang, Liaoning | Chancellor |
| L21 | 16-Jun-14 | Shenyang, Liaoning | Little Club nursery |
| H1-2-1 | 18-Jun-14 | Harbin, Heilongjiang | Longmai 26 |
| H1-2-2 | 18-Jun-14 | Harbin, Heilongjiang | Longmai 26 |
| H1-3-1 | 18-Jun-14 | Harbin, Heilongjiang | Longmai 26 |
| H1-5-1 | 18-Jun-14 | Harbin, Heilongjiang | Advanced Lines |
| H1-5-2 | 18-Jun-14 | Harbin, Heilongjiang | Advanced Lines |
| H1-5-3 | 18-Jun-14 | Harbin, Heilongjiang | Advanced Lines |
| H1-5-4 | 18-Jun-14 | Harbin, Heilongjiang | Little Club nursery |
| H1-5-5 | 18-Jun-14 | Harbin, Heilongjiang | Little Club nursery |
| H2-1 | 19-Jun-14 | Beian, Heilongjiang | Longken 3 |
| H2-1-4 | 19-Jun-14 | Beian, Heilongjiang | Longken 4 |
| H2-2-2 | 19-Jun-14 | Beian, Heilongjiang | Kehua (Parental nursery) |
| H2-2-3 | 19-Jun-14 | Beian, Heilongjiang | Kehua (Parental nursery) |
| H3-1-2 | 20-Jun-14 | Jiusan, Heilongjiang | Landrace wheat |
| H3-1-4 | 20-Jun-14 | Jiusan, Heilongjiang | Landrace wheat |
| H3-1-5 | 20-Jun-14 | Jiusan, Heilongjiang | Mingxian 169 (Trapping nursery) |
| H3-2-2 | 20-Jun-14 | Jiusan, Heilongjiang | Advanced Lines |
| H3-4-1 | 20-Jun-14 | Jiusan, Heilongjiang | Landrace wheat |
| H3-4-2 | 20-Jun-14 | Jiusan, Heilongjiang | Volunteer wheat |
| H3-4-3 | 20-Jun-14 | Jiusan, Heilongjiang | Advanced Lines |
| 09558-1 | 21-Jun-14 | Keshan, Heilongjiang | Ke94F4-408 |
| 09558-2 | 21-Jun-14 | Keshan, Heilongjiang | Ke94F4-408 |
| 09558-3 | 21-Jun-14 | Keshan, Heilongjiang | Kechun 6 |
| 09558-4 | 21-Jun-14 | Keshan, Heilongjiang | Local variety, breeding parent nursery |
| 09558-5 | 21-Jun-14 | Keshan, Heilongjiang | Local variety, breeding parent nursery |
| T2 | 7-Jun-14 | Tianshui, Gansu | CI14189 |
| T3-1 | 7-Jun-14 | Tianshui, Gansu | CI14189 |
| T3-1-1 | 7-Jun-14 | Tianshui, Gansu | Khapli/8cc |
| T3-1-2 | 7-Jun-14 | Tianshui, Gansu | Khapli/8cc |
| T4-1 | 9-Jun-14 | Tongwei, Gansu | Lantian 19 |
| T5-1 | 9-Jun-14 | Tongwei, Gansu | Lantian 19 |
| T7 | 9-Jun-14 | Tongwei, Gansu | Landrace wheat |
| G1-3-1 | 10-Jun-14 | Lintao, Gansu | Afu |
| G1-3 | 8-Jun-14 | Gangu, Gansu | Afu |
| G1-1-1 | 8-Jun-14 | Gangu, Gansu | Local variety, breeding parent nursery |
| G10 | 10-Jun-14 | Lintao, Gansu | Trapping nursery |
| G1-3-2 | 10-Jun-14 | Lintao, Gansu | Chancellor |
| G1-2 | 8-Jun-14 | Gangu, Gansu | Local variety, breeding parent nursery |
| G8 | 10-Jun-14 | Lintao, Gansu | Chancellor |
